# A new parasitic copepod (Copepoda; Cyclopoida; Chondracanthidae) from two pomacentrid fishes caught on the Great Barrier Reef, Queensland, Australia

**DOI:** 10.1007/s11230-022-10049-1

**Published:** 2022-06-29

**Authors:** Geoff A. Boxshall

**Affiliations:** Department of Life Sciences, Natural History Museum, Cromwell Road, London, SW7 5BD UK

## Abstract

A new species of the copepod genus *Pseudacanthocanthopsis* Yamaguti & Yamasu, 1959 (family Chondracanthidae) is described based on material of both sexes collected from two pomacentrid host fishes caught off Lizard Island, Queensland. The type host is *Neopomacentrus azysron* (Bleeker) and the additional host is *N. cyanomos* (Bleeker). The new species is distinguishable from all congeners by the form of the antennule of the female, which is dorso-ventrally flattened and extends out anteriorly to the front of the cephalothorax margin.

## Introduction

The copepod family Chondracanthidae comprises 191 valid species currently classified in 50 genera (Walter & Boxshall, 2021). Adult female chondracanthids are highly transformed parasites that live on marine fish hosts. The males are reduced in size and have been traditionally been referred to as dwarf males (Østergaard et al., 2005). Male chondracanthids are typically found attached to specialized secretory organs located in the genital region of the females from which they obtain nutrients (Østergaard & Boxshall, 2004).

The Chondracanthidae of Australian marine fishes are reasonably well known, with a total of 17 named species recorded to date (Table 1). The first two species reported were described by Heegaard (1940) under the names *Acanthochondria platycephali* Heegaard, 1940 and *Acanthochondria platycephali* forma *alata-longicollis* Heegaard, 1940. Both species were subsequently redescribed from the type material by Ho (1973) who accepted the former as valid and treated the latter as the type species of a new genus under the name *Pterochondria alatalongicollis* (Heegaard, 1940). In that paper, Ho (1973) considered that *Acanthochondria diastema* Kabata, 1965 described from waters off Tasmania was a junior synonym of *Pterochondria alatalongicollis*, but *A. diastema* was later resurrected as a valid species by Ho & Dojiri (1988). More recently, Tang et al. (2010) reassessed the validity of the genus *Pterochondria* Ho, 1973 and concluded that it did not differ significantly from *Acanthochondria*. They transferred its only species back to *Acanthochondria* as *A*. *alatalongicollis*.

**Table 1:** Species of Chondracanthidae known from Australian waters and their recorded hosts (listed in chronological order)

Copepod species	Host in Australian waters	References
*Acanthochondria platycephali* Heegaard, 1940	*Platycephalus bassensis* Cuvier	Heegaard (1940)
[as *A. gemina* Heegaard, 1962]	*Platycephalus richardsoni* Castlenau [as *Neoplatycephalus richardsoni*]	Heegaard (1962)
*Acanthochondria alatalongicollis* Heegaard, 1940	*Platycephalus bassensis* Cuvier	Heegaard (1940)
[as *Pterochondria alatalongicollis*]	*Platycephalus bassensis* Cuvier	Ho (1973)
*Acanthochondria tasmaniae* Heegaard, 1962	“sea perch”	Heegaard (1962)
*Pseudoblias lyrifera* Heegaard, 1962	*Rhombosolea tapirina* Günther	Heegaard (1962)
	*Pseudorhombus dupliciocellatus* Regan	Kabata (1969)
*Acanthochondria diastema* Kabata, 1965	*Platycephalus bassensis* Cuvier	Kabata (1965)
	*Platycephalus* sp.	Ho & Dojiri (1988)
*Neobrachiochondria quadrata* Kabata, 1969	*Hypoplectrodes nigroruber* (Cuvier)	Kabata (1969)
*Pseudacanthocanthopsis rohdei* Ho & Dojiri, 1976	*Dascyllus reticulatus* (Richardson)	Ho & Dojiri (1976)
	*Pomacentrus chrysurus* (Cuvier) [as *P*. *rhodonotus*]	Ho & Dojiri (1976)
*Lagochondria nana* Ho & Dojiri, 1988	*Callionymus* sp.	Ho & Dojiri (1988)
*Apodochondria medusa* Ho & Dojiri, 1988	*Neosebastes pandus* (Richardson)	Ho & Dojiri (1988)
*Acanthochondria incisa* Shiino, 1955	*Scorpaena papillosa* (Schneider & Forster) [as *Helicolenus papillosus*]	Kabata (1992)
*Chondracanthus genypteri* Thomson, 1899	*Genypterus blacodes* (Forster)	Kabata (1992)
*Chondracanthus neali* Leigh-Sharpe, 1930	*Malacocephalus laevis* (Lowe)	Kabata (1992)
*Chondracanthus polymixiae* Yamaguti, 1939	*Polymixia japonica* Günther	Kabata (1992)
*Rohdea cryptopoda* Kabata, 1992	*Genypterus blacodes* (Forster)	Kabata (1992)
*Lateracanthus novus* Kabata, 1992	*Cetonurus* sp.	Kabata (1992)
*Acanthocanthopsis quadrata* Heegaard, 1945	*Dicotylichthys punctulatus* Kaup	Tang & Ho (2005)
	*Contusus brevicaudus* Hardy	Tang & Ho (2005)
*Chondracanthus goldsmidi* Tang, Andrews & Cobcroft, 2007	*Latris lineata* (Forster)	Tang et al. (2007)
Chondracanthidae gen .sp.	*Paraulopus nigripinnis* (Günther) [as *Chlorophthalmus nigripinnis*]	Kabata (1992)

Heegaard (1962) added *Pseudoblias lyrifera* Heegaard, 1962 to the Australian fauna and this species was redescribed in better detail by Kabata (1969). In the same paper Kabata established *Neobrachiochondria quadrata* Kabata, 1969, a new monotypic genus from southern Australia. Ho & Dojiri (1976) described *Pseudacanthocanthopsis rohdei* Ho & Dojiri, 1976 from the Great Barrier Reef, and this is the only species reported from Australian waters on a pomacentrid host, the same host family as the new species described below. Two major contributions to our knowledge came from Ho & Dojiri (1988) who established two new monotypic genera, *Lagochondria* Ho & Dojiri, 1988 and *Apodochondra* Ho & Dojiri, 1988, from Australian marine fishes, and Kabata (1992) who recorded six species new to Australia, including the new monotypic genus *Rohdea* Kabata, 1992, collected from fishes caught in deeper waters off the coast of New South Wales. These existing records are summarized in Table 1.

Here, we describe a new species of *Pseudacanthocanthopsis* Yamaguti & Yamasu, 1959 collected from two pomacentrid hosts caught from small reefs in the lagoon and in front of Casuarina Beach off Lizard Island, Queensland (Great Barrier Reef, 14°400′S, 145°280′E). For map see Grutter (1996). A third pomacentrid species, *Pomacentrus moluccensis* Bleeker, 1853, collected at the same time, in the same location, and using the same methods was uninfected by the parasite described here (Grutter, 1996).

## Materials and methods

The material was collected by Alexandra S. Grutter as part of a field experiment testing the effect of cleaner fish *Labroides dimidiatus* Valenciennes presence/removal on ectoparasites (Grutter, 1996). Fish were collected by scuba divers using barrier nets and hand nets, and immediately placed in quick-sealing plastic bags to retain the parasites. Fish died when placed in an ice slurry during transport to the laboratory. The fish and the contents of the bag were fixed in 10% formalin in seawater. Parasites were found by examining the body of the fish, oral and branchial cavities, inside of operculum, and detached pectoral and pelvic fins and gills spread out on a petri dish, all scanned under a microscope at a magnification of 35x. Collected parasites were transferred to individual vials containing 10% formalin in seawater. Fish standard length (SL) and total length (TL) were measured.

Three females of *Pseudacanthocanthopsis* were collected from the gills of *Neopomacentrus azysron* (Bleeker, 1877) caught between October 18 and 26, 1993. A further three females of *Pseudacanthocanthopsis* were collected from the gills of *Neopomacentrus cyanomos* (Bleeker, 1856) caught between October 18 and 19, 1993. Specimens were cleared in lactic acid and observed whole on a Leitz dissecting microscope. Dissected appendages were examined on an Olympus BH2 microscope using differential interference contrast. Drawings were made using a drawing tube and measurements were made using a stage micrometer. Morphological terminology conforms to Huys & Boxshall (1991). The structure referred to in older chondracanthid literature as the “accessory antennule” was shown to be the atrophied (and laterally displaced) terminal segment of the antenna (Ho, 1984). Names of hosts follow FishBase (Froese & Pauly, 2021). Type material and voucher specimens are deposited in the collections of the Queensland Museum and in the Natural History Museum, London.

## Systematics

Family Chondracanthidae Milne Edwards, 1840

Genus *Pseudacanthocanthopsis* Yamaguti & Yamasu, 1959

***Pseudacanthocanthopsis grutterae*** **sp. nov.**

Type Material: Holotype female from gills of *Neopomacentrus azysron* (Fish No. 3023: 35.8 mm SL, 44.4 mm TL); Queensland Museum Registration No. W29609. Paratype female with male attached, from gills of *N. azysron* (Fish No. 3430: 42.9 mm SL, 51 mm TL); Queensland Museum Registration No. W29610. Paratype female (without male), from gills of *N. azysron* (Fish No. 3114: 55.2 mm SL, 73.8 mm TL); Natural History Museum, London Registration number NHMUK 2022.172.

Additional non-type material: one female with male attached, from gills of *Neopomacentrus cyanomos* (Fish 3082: 42.6 mm SL, 52 mm TL); Queensland Museum Registration No. W29611. One female with male attached, from gills of *N. cyanomos* (Fish 3065: 34.4 mm SL, 42.6 mm TL); Queensland Museum Registration No. W29612. One female with male attached, from gills of *N. cyanomos* (Fish 3009: 39.8 mm SL, 49.2 mm TL); Natural History Museum, London Registration number NHMUK 2022.173.

Abundances (range, median, 25th/75th quantile, prevalence, number of fish hosts sampled) were: *Neopomacentrus azysron* (0 – 4, 0, 0/0, 17.8%, 135); *Neopomacentrus cyanomos* (0-6, 0, 0/2, 49%, 104) (Unpublished data, Alexandra S. Grutter, personal communication).

### Description of Female

**Fig. 1:**
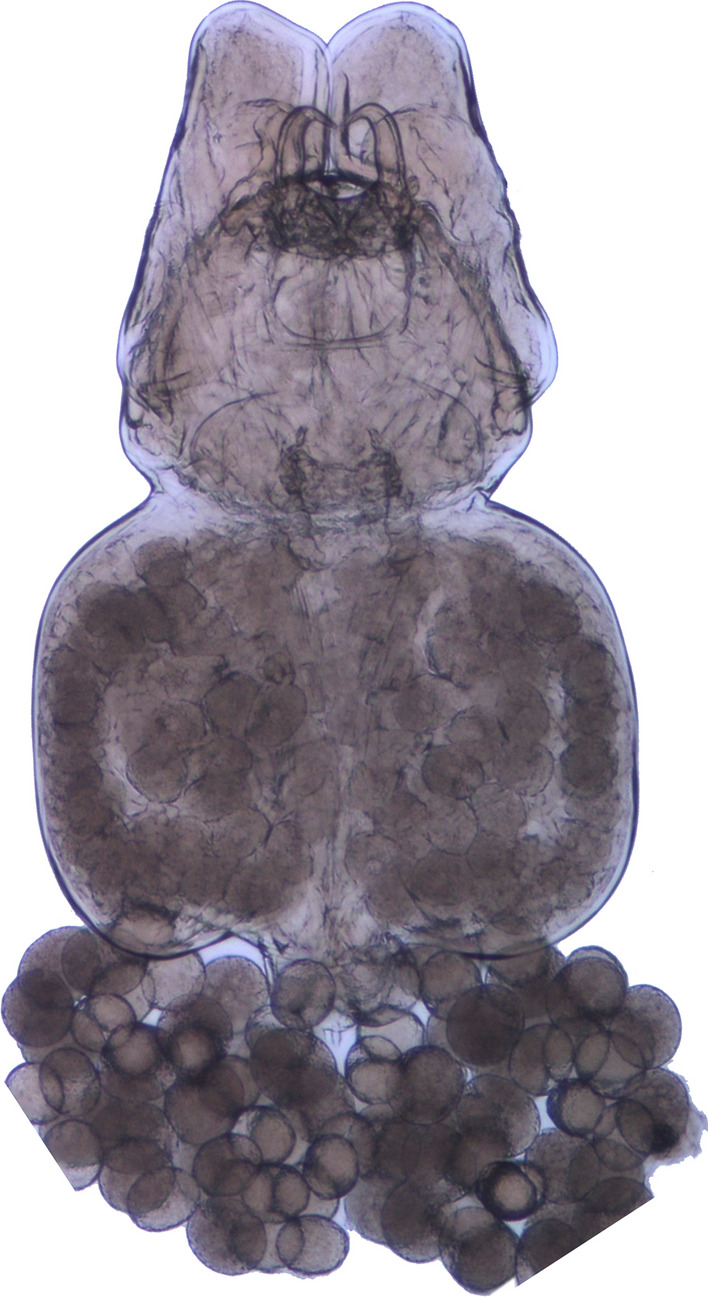
Photomicrograph of adult female of *Pseudacanthocanthopsis grutterae* **n. sp.** (ventral view) from *Neopomacentrus cyanomos*

**Fig. 2:**
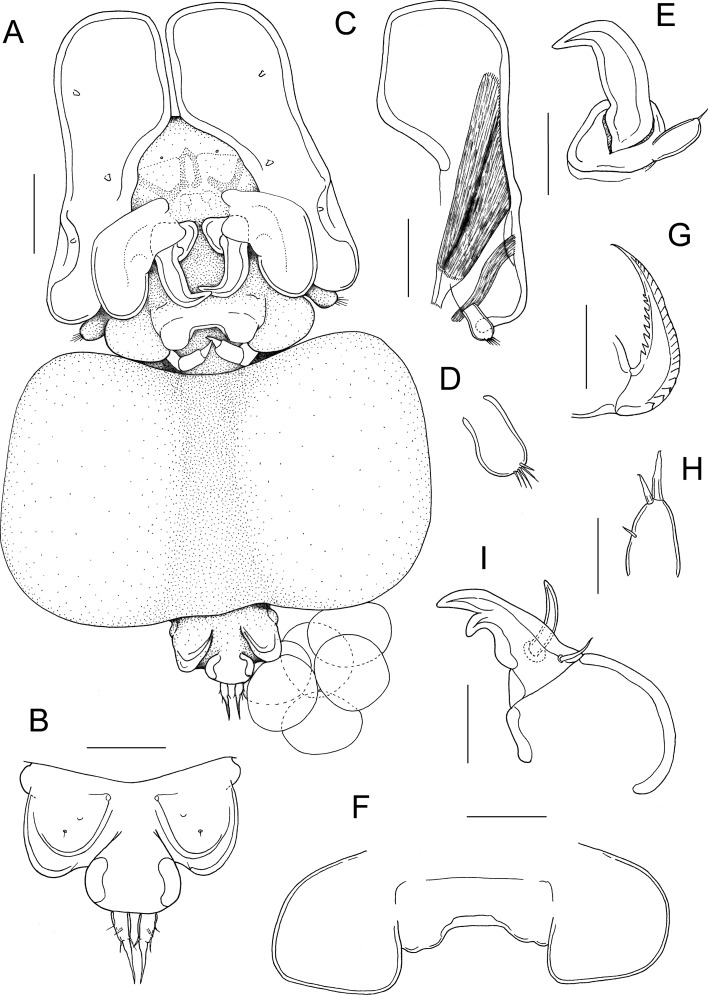
*Pseudacanthocanthopsis grutterae* **n. sp.** adult female. A, habitus, ventral; B, genitoabdomen, dorsal; C, antennule showing musculature; D, distal segment of antennule; E, antenna; F, labrum *in situ*, ventral; G, mandible; H, maxillule; I, maxilla. Scale bars: A, C, 100 μm; B, E, F, 50 μm; H, I, 20 μm; G, 10 μm

**Fig. 3:**
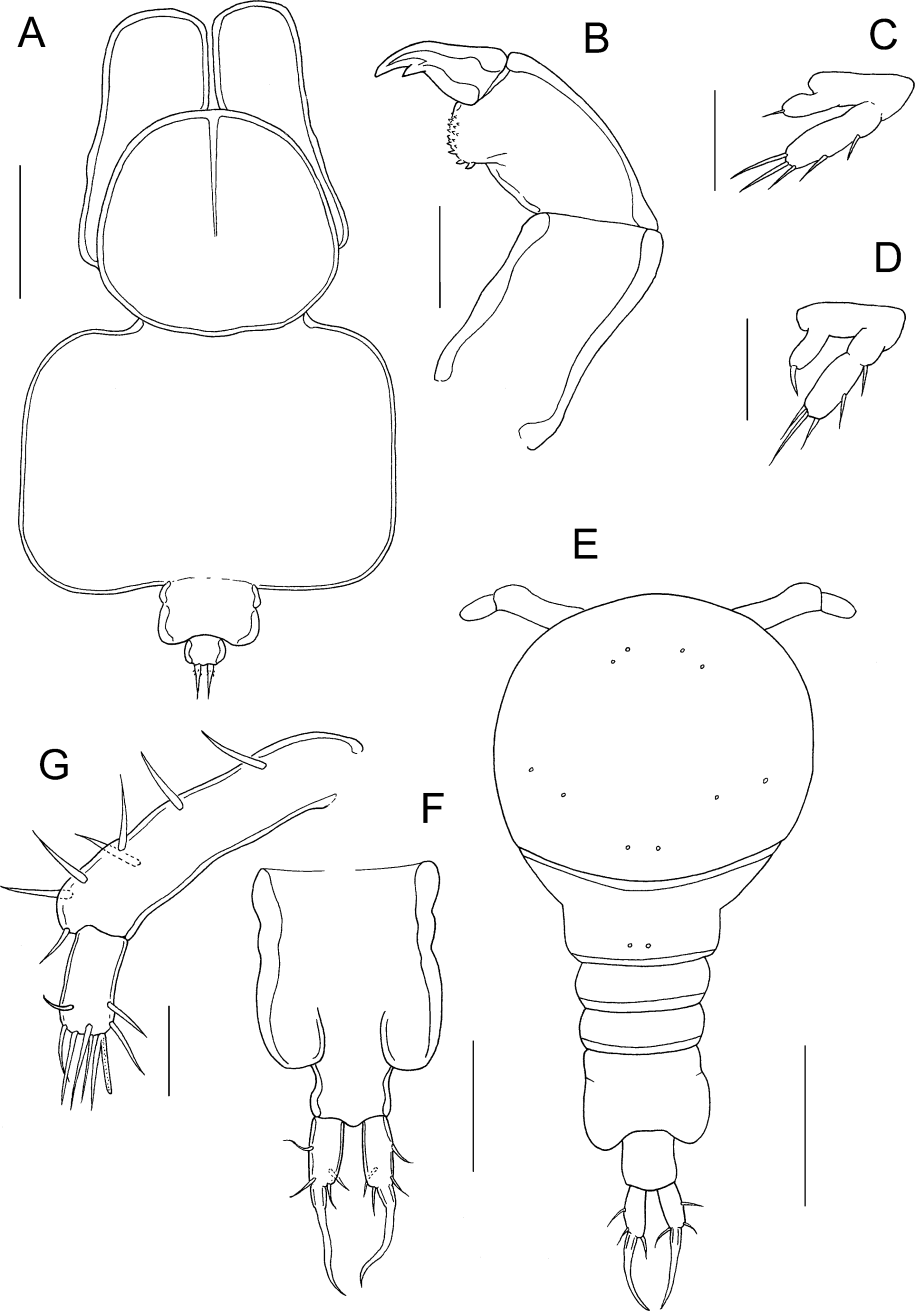
*Pseudacanthocanthopsis grutterae* **n. sp.** adult female. A, habitus, dorsal; B, maxilliped; C, leg 1; D, leg 2. Adult male, E, habitus, dorsal; F, genital complex and abdomen, ventral; G, antennule. Scale bars: A, 200 μm, B-D, 20 μm; E, 50 μm; F, 25 μm; G, 10 μm

Adult female body transformed, consisting of head, trunk and genitoabdomen (Figs. 1, 2A, 3A); total body length 868 μm (excluding antennules and caudal setae). Head comprising fused cephalosome and first pedigerous somite; slightly wider than long (347 x 362 μm); with median cuticular thickening along dorsal midline extending posteriorly from frontal margin to beyond middle of head (Fig. 3A). Proximal segment of antennules flattened and extending anteriorly to form bipartite frontal plate, conspicuous in dorsal view. Head without obvious dorsal or lateral processes. Paired processes associated with antennae present; processes and antennae can together be displaced anteriorly (Fig. 1) or posteriorly (Fig. 2A) in preserved material. Small posterolateral rounded lobes present ventrally either side of labrum. Trunk about 1.5 times wider than long (370 x 574 μm), subrectangular, lacking processes. Genitoabdomen (Fig. 2B) small, comprising genital complex bearing paired genital openings dorsolaterally, and unsegmented abdomen. Genital complex about 2.2 times wider than abdomen; ornamented with paired setules on ventral surface. Abdomen about as wide as long, fused to genital complex, bearing paired caudal rami on posterior margin. Caudal rami each armed with large fused apical seta plus 1 inner distal seta, 1 dorsal seta and 2 lateral setae.

Antennule (Fig. 2C, D) 2-segmented, comprising proximal segment forming enlarged dorso-ventrally flattened plate and cylindrical distal segment. Proximal segment expanded anteriorly, supplied with extrinsic muscles entering via lumen at base of limb. Three stubby elements (modified setae?) present on ventral surface of proximal segment (Fig. 2A). Distal segment (Fig. 2D) displaced posteriorly, armed with 5 setae apically.

Antenna (Fig. 2E) comprising broad compound basal segment and strongly curved distal claw; atrophied terminal segment (“accessory antennule”) located laterally at base of claw; armed with single apical seta. Labrum (Fig. 2F) with median indentation and expanded into paired lateral lobes. Mandible (Fig. 2G) forming tapering blade armed with about 22 teeth along convex margin and 8 on concave margin. Maxillule (Fig. 2H) lobate with 2 apical setae and 1 reduced inner seta. Maxilla (Fig. 2I) 2-segmented, proximal segment robust, unarmed; distal segment forming stout curved claw bearing strong accessory claw on concave margin; and armed with curved spiniform seta plus slender seta proximally. Maxilliped Fig. 3B) 3-segmented; first segment longest, unarmed; second segment with inner distal margin produced into swelling ornamented with fine spinules; terminal segment forming curved claw bearing small tooth on concave margin.

Legs 1 (Fig. 3C) and 2 (Fig. 3D) located on walls of groove separating head and trunk; difficult to observe. Both leg pairs biramous but very reduced, each comprising unsegmented protopodal part, unsegmented, lobate endopod and indistinctly 2-segmented exopod. Endopod with single apical seta in both legs. Exopod armed with outer element on proximal segment and 1 lateral plus 3 apical elements on distal segment in both legs.

### Description of Male

Body less transformed than female; consisting of cephalothorax (comprising fused cephalosome plus first pedigerous somite), second pedigerous somite, 2 limbless somites (expressed in dorsal view), genital complex and 1-segmented abdomen (Fig. 3E). Cephalothorax slightly wider than long (91 x 100 μm); surfaced ornamented with 5 pairs of integumental pores. Second pedigerous somite narrowing strongly; posterior part ornamented with pair of pores dorsally. Third somite limbless; wider than long (14 x 42 μm). Fourth somite clearly expressed dorsally (Fig. 3E) but fully fused to genital complex ventrally (Fig. 3F); dimensions in dorsal view (13 x 39 μm). Genital complex (Fig. 3E) with slight lateral indentation marking plane of fusion of fifth somite with genital somite; produced posterolaterally into rounded lobes carrying genital apertures ventrally. Abdomen as long as wide (17 x 17 μm); bearing paired caudal rami posteriorly. Caudal rami (Fig. 3F) about twice as long as wide (13 x 6 μm); armed with large fused apical seta plus 1 inner distal seta, 1 dorsal seta and 2 lateral setae.

**Fig. 4:**
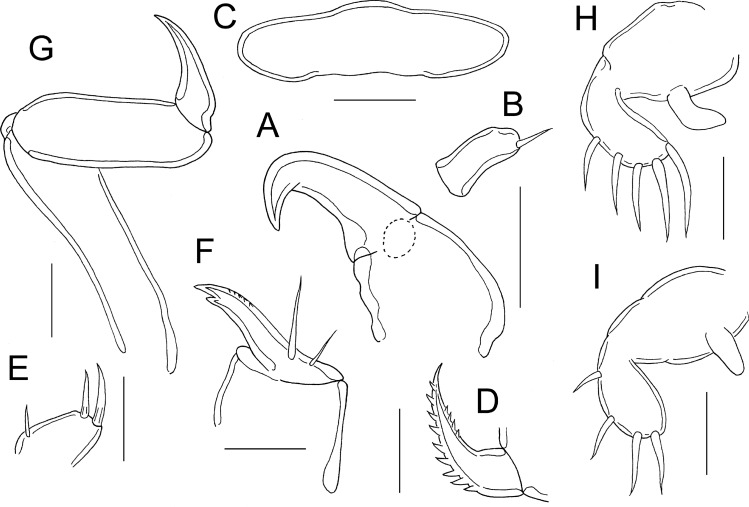
*Pseudacanthocanthopsis grutterae* **n. sp.** adult male. A, antenna, with position of atrophied distal segment marked; B, atrophied apical segment of antenna, detached; C, labrum *in situ*, ventral; D, mandible; E, maxillule; F, maxilla; G, maxilliped; H, leg 1; I, leg 2. Scale bars: A, B, 20 μm; C-I, 10 μm

Antennule (Fig. 3G) cylindrical, 2-segmented; large basal segment armed with 7 setae along anteroventral surface; apical segment short, armed with 8 setae in total, one of which fused basally with aesthetasc. Antenna (Fig. 4A, B) comprising large, unarmed basal segment bearing strongly recurved terminal claw; atrophied apical segment (“accessory antennule”) carried on lateral surface; apical segment (Fig. 4B) cylindrical, armed with single apical seta. Labrum (Fig. 4C) without median indentation on posterior margin; with rounded lateral margins. Mandible (Fig. 4D) forming tapering blade armed with 9 teeth along convex margin and 4 on concave margin. Maxillule (Fig. 4E) lobate with 2 apical setae and 1 reduced inner seta. Maxilla (Fig. 4F) 2-segmented; proximal segment robust, unarmed; distal segment forming weakly curved claw bearing strong tooth distally on concave margin and 5 denticles on outer margin; segment armed with 2 slender setae proximally. Maxilliped (Fig. 4G) slender; first segment elongate, unarmed; second segment unarmed; third segment forming simple, tapering distal claw.

Legs 1 and 2 (Fig. 4H, I) biramous, reduced; each comprising unsegmented protopod, unsegmented, lobate endopod and indistinctly segmented exopod. Endopod unarmed in both legs. Exopod armed with 5 outer and distal margin elements in leg 1 (Fig. 4H) and 4 outer and distal margin elements in leg 2 (Fig. 4I).

Etymology. The new species honours its discoverer Alexandra S. Grutter for her important contributions to our knowledge of the ectoparasites of Great Barrier Reef fishes and the impact of cleaner fish on these communities.

### Remarks

The genus *Pseudacanthocanthopsis* was established by Yamaguti & Yamasu (1959) to accommodate their new species *P. apogonis* Yamaguti & Yamasu, 1959, collected from *Ostorhinchus semilineatus* Temminck & Schlegel [as *Apogon semilineatus*] in Japanese waters. The type species was subsequently reported from a second apogonid host*, Ostorhinchus doederleini* Jordan & Snyder [as *Apogon doederleini*] (Izawa, 1975). Two additional species were subsequently reported from Japan. The first was *P. secunda* Yamaguti & Yamasu, 1960 from *Apogon lineatus* Temminck & Schlegel. This species was described under the name *Pseudacanthopsis secunda*: however, this generic name has never been proposed and is obviously an error since Yamaguti and Yamasu (1960) only made comparisons with *Pseudacanthocanthopsis apogonis*. Venmathi Maran et al. (2013) redescribed *P. secunda* and recorded its presence on two new hosts: the tetraodontid *Takifugu poecilonotus* (Temminck & Schlegel) caught off Hiroshima Prefecture, Japan, and the sparid *Pagrus major* (Temminck & Schlegel) from the Seto Inland Sea, Japan. They also extended its known distribution range on the type host, *A. lineatus*, to include the East China Sea off Japan and Korea. The third Japanese species was *P. bicornutus* (Shiino, 1960) from a cepolid host, *Owstonia totomiensis* Tanaka. Ho & Kim (1995) redescribed *P. bicornutus* based on material from a second host, the pomacentrid *Chromis notatus* (Temminck & Schlegel), caught in the Sea of Japan. The fourth species in the genus is *P. rohdei* which was based on material collected from two hosts belonging to the family Pomacentridae, *Dascyllus reticulatus* (Richardson) and *Pomacentrus chrysurus* (Cuvier) [as *P*. *rhodonotus* Bleeker] in Australian waters (Ho & Dojiri, 1976).

The new species can be readily distinguished from all four of its congeners by the form of the antennule in females, which is dorso-ventrally flattened and extends out anteriorly to the front of the cephalothorax. Together the antennules are visible as a bifid plate in dorsal view (Fig. 3A). In contrast, in the three Japanese species, *P. apogonis, P. secunda* and *P. bicornutus*, the antennules of the female are cylindrical and more-or-less directed laterally. In *P. rohdei* the antennules are modified, with the large, fleshy proximal segment forming a curved structure with a ventromedial lobe and a large protrusion at its posterodistal corner (Ho & Dojiri, 1976). However it is fleshy and cylindrical rather than dorso-ventrally flattened. The unique form of the antennule of the new species is observable in undissected females and supports the establishment of the new species.

The morphology of the new species is most similar to that of *P. rohdei* and *P. bicornutus*; all three species have very similar gross morphology in the female and the appendages share numerous character states. For example, all three species have a reduced number of setae on the atrophied tip of the antenna, an accessory claw on the maxillary basis, a spinulose lobe on the basis of the maxilliped, subequal legs 1 and 2, and a reduced number of setae on the endopod of both legs in the adult female; and lack a strong claw on the atrophied tip of the antenna and have a reduced number of setae on the endopod of leg 2 in the adult male. In addition to the antennules, the new species differs from *P. rohdei* and *P. bicornutus* by having a large fleshy process near the base of each antenna, one seta (vs. none) on the atrophied tip of the antenna, no tooth on the maxillary basis (vs. one small tooth – see Fig. 5D in Ho & Dojiri (1976) for *P. rohdei* and Fig. 6A in Ho & Kim (1995) for *P. bicornutus*), four setae (vs. five setae) on the distal exopodal segment of leg 1, one seta (vs. two setae) on leg 1 endopod and four setae (vs. 3 or 5 setae) on the distal exopodal segment of leg 2 in the female; and by having one seta (vs. 3 or 5 setae) on the atrophied tip of the antenna, and no setae (vs. 2 or 4 setae) on the endopod of legs 1 and 2 in the male.

## Discussion

The form of the female antennules in the new species is so unusual that investigation of the musculature was necessary in order to confirm the interpretation. Two extrinsic muscles pass into the antennule from the head (Fig. 2C). The larger, more anteriorly located muscle has a tendinous origin inside the head and passes into the proximal segment of the antennule and on towards a broad insertion on the lateral wall of the segment. The smaller muscle also originates inside the head and passes into the limb, inserting directly onto the lateral wall of the segment posterior to the anterior muscle. The presence of musculature indicates that the flattened structure is derived from paired limbs and the presence of the defined, setose, distal segment, albeit displaced posteriorly, confirms that this structure comprises the proximal part of the antennule. These muscles may represent an opposing pair and appear to function to adduct and abduct the flattened proximal segment relative to the head, possibly moving the anterior expansions in to meet at the midline and to separate them.

Ho & Dojiri (1976) noted that the “pygmy” male of *P. rohdei* attached to one of a pair of small processes found at the junction of the trunk and the genital complex of the adult female. No such processes were observed in the new species but it is clear that the structures reported by Ho & Dojiri (1976) represent the paired nuptial organs. Østergaard & Boxshall (2004) interpreted these specialized structures as secretory organs which provide nutrient secretion that sustains the attached male.

## Data Availability

Type material is stored in the Queensland Museum and Natural History Museum, London and is available for study.
